# Inhalational Wheat-pill Poisoning: A Household Chemical Warfare Agent

**DOI:** 10.7759/cureus.5597

**Published:** 2019-09-08

**Authors:** Zauraiz Anjum, Muhammad N Habib, Zemal Tariq, Shaharyar Ali

**Affiliations:** 1 Internal Medicine, Fatima Jinnah Medical University, Lahore, PAK; 2 Internal Medicine, King Edward Medical University/Mayo Hospital, Lahore, PAK; 3 Internal Medicine, Gujranwala Medical College, Gujranwala, PAK

**Keywords:** inhalational, poisoning, aluminum phosphide, phosphene gas, wheat pill

## Abstract

Wheat pill and its active ingredient aluminum phosphide is one of the emerging causes of poisoning. The areas mainly hit are the ones where agriculture is the leading industry. The mechanism of action involves phosphine gas, which is released when the pill comes in contact with moisture or stomach acid; the resulting free radicals damage mitochondria. The cardiovascular system is the most severely hit with various presentations, including cardiac failure, arrhythmias, and eventually shock. We discuss a case of a middle-aged woman who suffered from inhalational poisoning from wheat pill while she was working in unventilated grain storage.

## Introduction

Wheat pill or rice pill is widely used as a rodenticide in agriculture-based communities [1]. The mechanism of action has phosphine gas as the final culprit that causes free radical damage to mitochondria [2-3]. Wheat-pill poisoning leads to catastrophic sequelae, with the main target being the heart, which results in arrhythmias, cardiac failure, and eventually refractory shock and multiorgan dysfunction syndrome [2]. Although cases of orally ingested wheat pills for suicide and poisoning by fumigation are prevalent [1], we hereby discuss the case of wheat-pill poisoning due to inhalation in a closed space. In an extensive review of literature on PubMed using the keywords “wheat pill,” “aluminum phosphide” and “poisoning,” we could find only one reported case of poisoning by aluminum phosphide due to inhalation in a closed space [4]. The objective of this article is to inform physicians about this unique mode of poisoning so that timely and appropriate management can be commenced to avoid an unfavorable outcome [5].

## Case presentation

A 44-year-old female presented to the emergency department with complaints of nausea, vomiting, abdominal pain, frothing from the mouth, and marked agitation. On inquiry, her family members reported that she was in her usual state of health four hours ago when she started to clean the moist wheat storage unit. The patient denied any accidental or intentional intake of ‘suspicious’ materials in the last 24 hours. She denied any: headache, vertigo, shortness of breath, cough, chest pain, or any history of recent travel or insect bite. Her past medical, surgical, allergic, gynecological, social, and sexual history was non-contributory.

On examination, she appeared agitated and was sweating. She had no bite marks on her body. Her pulse was 110/min and regular, blood pressure was 110/70 mmHg, respiratory rate was 22/min, blood sugar level (BSL) was 110 mg/dL. Oxygen saturation was 88%. Her Glasgow Coma Scale (GCS) was E4, V4, M6. Her motor and sensory system was intact as were her cranial nerves. The chest was clear to auscultation bilaterally. Cardiac auscultation revealed tachycardia with no added sounds. The abdomen was tender, and bowel sounds were normal.

Investigations

Arterial blood gas (ABG) showed partially compensated metabolic acidosis. Electrocardiogram (ECG) showed occasional ectopic ventricular beats. The rest of the initial blood work was normal.

Management and outcome

**Figure 1:**
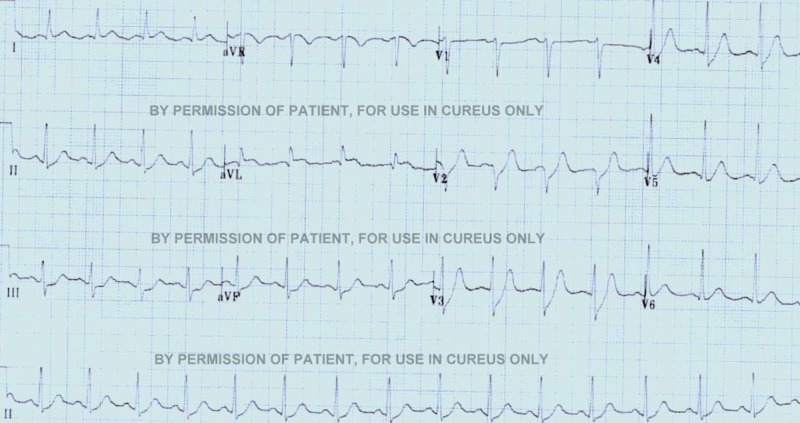
The second ECG of the patient with ST-depressions in Inferior leads.

The patient was admitted to the intensive care unit (ICU), supplemental oxygen was given by facemask, and two wide-bore intravenous (IV) lines were saved. Cardiac, peripheral capillary oxygen saturation (SpO2), and blood pressure monitors were continuously attached, and BSL monitoring was done every half hour. A Foley catheter was placed to monitor the input and output of fluids. After one hour, her SpO2 fell to 93% on supplemental oxygen, blood pressure fell to 80/60 mmHg, and BSL was found to be 86 mg/dL. We started IV normal saline and 5% dextrose water. Her ECG showed ST-depression mainly in the inferior leads (Figure 1).

Over the next 6 hours, her blood pressure continued to drop, and we started inotropic and vasotropic support (dobutamine and norepinephrine) at maximum doses alongside IV normal saline. A central venous line was placed to measure the central venous pressure for guiding fluid management. Her saturation fell to 86%, and the patient was intubated and put on ventilator support. Her repeat labs showed worsening metabolic acidosis, but pH remained above the cut-off for bicarbonate replacement. Her blood urea nitrogen (BUN) was 70 mg /dl, creatinine (Cr) 3.3 mg/dl, aspartate aminotransferase (AST) 400, alanine aminotransferase (ALT) 550, creatine kinase-muscle/brain (CK-MB) 330, sodium (Na) 126, potassium (K) 3.2, calcium (Ca) 7.3.

Despite our maximum efforts, she continued to deteriorate over the next 12 hours with progressively worsening labs. The patient had developed refractory shock and multiorgan dysfunction. She could not be resuscitated and passed on.

## Discussion

The wheat pill is a well-known rodenticide in agricultural areas. In an article reviewing the cases of aluminum phosphide poisoning in Saudi Arabia for over nine years, the authors found that 68 patients presented with the poisoning, among which 38 were female. 22 people died, and 18 of them were below 20 years of age [1].

Its chemical formula contains aluminum phosphide in addition to other chemicals. Aluminum phosphide releases phosphine gas when it comes in contact with moisture. The gas, in turn, does free radical damage to mitochondria [2-3].

The signs and symptoms of the poisoning encompass a multitude of organ systems. They broadly include agitation, occasional seizures, eventual coma, dyspnea, pulmonary edema, heart failure, arrhythmias, ECG abnormalities, abdominal pain, nausea, vomiting, oliguria, metabolic acidosis, hyponatremia, hypo/hyperkalemia, hypomagnesemia, and hypocalcemia [2].

The complications and sequelae of the poisoning include cardiac failure, hepatic failure, pancreatitis, renal failure, and circulatory and cardiogenic shock [2]. The main target seems to be the cardiovascular system so much so that, sometimes, the poisoning can be confused with a cardiac ischemic event [6]. A rare complication has been described as intravascular hemolysis [7].

The diagnosis can be furnished by a thorough history and examination and the silver nitrate test. Moreover, the serum levels of phosphine gas have high sensitivity [2]. Another article reviewed favorably the headspace gas chromatography coupled with mass spectrometry (HS-GC-MS). According to the authors, it could measure even minute quantities of phosphine gas [8].

Although there is no specific antidote for the poisoning, various management strategies have been tested with varying degrees of success. A reduction in mortality has been seen with gastric lavage with potassium permanganate and mineral or coconut oil [9]. Other articles spoke favorably about other antidotes, including triiodothyronine, acetyl-L-carnitine, glutathione, liothyronine, milrinone, Laurus nobilis L, vasopressin, sodium selenite, sulfate, N-acetylcysteine, 6-aminonicotinamide, boric acid, melatonin, vitamins C and E [10], selegiline [11], and whole blood transfusion [12]. Other articles reviewed the roles of digoxin [13], extracorporeal cardiopulmonary resuscitation [14], aortic balloon [15], and prophylactic amiodarone for symptomatic improvement in cardiac function [16]. In another article, the authors discussed the protective role of G6PD deficiency in wheat pill poisoning [17].

Although aluminum phosphide poisoning is commonly seen in fumigation and via an oral route through wheat-pill ingestion, our patient accidentally got exposed to the poison while working in a damp, unaerated wheat storage unit. Our review of articles showed only one such reported case [4]. Through this article, we would like to bring to attention this rare mode of poisoning so that timely measures can be taken to avoid an unfavorable outcome, as studies have shown that timely intervention can save lives [5] especially in inhalational poisoning of aluminum phosphide [4].

## Conclusions

Wheat-pill poisoning (aluminum phosphide poisoning, phosphine gas) poisoning is quite common after ingestion or fumigation. Here, we discussed a case of a person getting aluminum phosphide poisoning after working in unventilated grain storage. Our review found only one other such case, and we want to highlight and bring this rare presentation to the physicians so that appropriate measures can be taken quickly to prevent mortality.

## References

[1] Alnasser S, Hussain SM, Kirdi TS, Ahmed A (2018). Aluminum phosphide poisoning in Saudi Arabia over a nine-year period. Ann Saudi Med.

[2] Ghazi MA (2013). “Wheat pill (aluminum phosphide) poisoning”; commonly ignored dilemma. A comprehensive clinical review. Professional Med J.

[3] Moghadamnia AA (2012). An update on toxicology of aluminum phosphide. Daru.

[4] Çakın Ö, Tazegul G, Gümüş A, Cengiz M, Ramazanoğlu A (2018). Incidental aluminum phosphide poisoning: case report and current management. Folia Med (Plovdiv).

[5] Hena Z, McCabe ME, Perez MM, Sharma M, Sutton NJ, Peek GJ, Clark BC (2018). Aluminum phosphide poisoning: Successful recovery of multiorgan failure in a pediatric patient. Int J Pediatr Adolesc Med.

[6] Ghosh S, Biswajit M, Chatterjee PK, Saurabh S, Sudeep KN, Shukla P, Sharmistha C (2018). Aluminum phosphide poisoning presenting like acute myocardial infarction in a young girl. J Assoc Physicians India.

[7] Malakar S, Negi BD, Dutt K, Raina S (2019). Intravascular hemolysis in aluminum phosphide poisoning. Indian J Crit Care Med.

[8] Yan H, Xiang P, Zhang S, Shen B, Shen M (2017). Diagnosis of aluminum phosphide poisoning using a new analytical approach: forensic application to a lethal intoxication. Int J Legal Med.

[9] Shadnia S, Rahimi M, Pajoumand A, Rasouli MH, Abdollahi M (2005). Successful treatment of acute aluminium phosphide poisoning: possible benefit of coconut oil. Plum Exp Toxicol.

[10] Karimani A, Mohammadpour AH, Zirak MR, Rezaee R, Megarbane B, Tsatsakis A, Karimi G (2018). Antidotes for aluminum phosphide poisoning - an update. Toxicol Rep.

[11] Maleki A, Hosseini MJ, Rahimi N (2019). Adjuvant potential of selegiline in treating acute toxicity of aluminium phosphide in rats. Basic Clin Pharmacol Toxicol.

[12] Zamani N, Hassanian-Moghaddam H, Ebrahimi S (2018). Whole blood exchange transfusion as a promising treatment of aluminium phosphide poisoning. Arh Hig Rada Toksikol.

[13] Changal KH, Latief M, Parry M, Abbas F (2017). Aluminium phosphide poisoning with severe cardiac dysfunction and the role of digoxin. BMJ Case Rep.

[14] Lehoux J, Hena Z, McCabe M, Peek G (2018). Aluminium phosphide poisoning resulting in cardiac arrest, successful treatment with extracorporeal cardiopulmonary resuscitation (ECPR): a case report. Perfusion.

[15] Mehrpour O, Asadi S, Yaghoubi MA, Azdaki N, Mahmoodabadi N, Javadmoosavi S (2019). Cardiogenic shock due to aluminum phosphide poisoning treated with intra-aortic balloon pump: a report of two cases [EPub]. Cardiovasc Toxicol.

[16] Beyranvand MR, Farrokhi S, Peyvandi H, Soltaninejad K, Shadnia S (2019). The effects of amiodarone prophylaxis on cardiac dysrhythmia in acute aluminium phosphide poisoning. Arh Hig Rada Toksikol.

[17] Humayun M, Haider I, Badshah A, Subhan F (2015). Protective role of G6PD deficiency in aluminium phosphide poisoning. J Coll Physicians Surg Pak.

